# Poly[oligo(ethylene glycol) methacrylate]-*b*-poly[(vinyl benzyl trimethylammonium chloride)] Based Multifunctional Hybrid Nanostructures Encapsulating Magnetic Nanoparticles and DNA

**DOI:** 10.3390/polym12061283

**Published:** 2020-06-03

**Authors:** Angeliki Chroni, Aleksander Forys, Barbara Trzebicka, Adam Alemayehu, Vaclav Tyrpekl, Stergios Pispas

**Affiliations:** 1 Theoretical and Physical Chemistry Institute, National Hellenic Research Foundation, 48 Vassileos Constantinou Avenue, 11635 Athens, Greece; angelikechrone@gmail.com; 2 Centre of Polymer and Carbon Materials, Polish Academy of Sciences, 34 ul. M. Curie-Skłodowskiej, 41-819 Zabrze, Poland; aforys@cmpw-pan.edu.pl (A.F.); barbara.trzebicka@cmpw-pan.edu.pl (B.T.); 3 Department of Inorganic Chemistry, Faculty of Science, Charles University in Prague, Albertov 6, 128-43 Praha 2, 11636 Prague, Czech Republic; adam.alemayehu@natur.cuni.cz (A.A.); vaclav.tyrpekl@natur.cuni.cz (V.T.)

**Keywords:** double-hydrophilic block copolymers, theranostics, cationic polyelectrolytes, magnetic nanoparticles, gene delivery, hybrid nanostructures

## Abstract

We report on the preparation of novel and multifunctional hybrid spherical-shaped nanostructures involving a double-hydrophilic block copolymer, namely the neutral cationic poly[oligo(ethylene glycol) methacrylate]-*b*-poly[(vinyl benzyl trimethylammonium chloride)] (POEGMA-*b*-PVBTMAC) diblock copolymer, initially complexed with hydrophilic anionic magnetic nanoparticles (MNPs), and subsequently, with short deoxyribonucleic acid (113 bases DNA). The POEGMA-*b*-PVBTMAC copolymer, the copolymer/MNPs and the copolymer/MNPs/DNA tricomponent hybrid electrostatic complexes were studied by dynamic/electrophoretic light scattering (DLS/ELS) and cryogenic transmission electron microscopy (cryo-TEM) techniques for the determination of their structure and solution properties. The MNPs were complexed efficiently with the oppositely charged diblock chains, leading to well-defined hybrid organic–inorganic spherical-shaped nanostructures. A significant aggregation tendency of the MNPs is noticed in cryo-TEM measurements after the electrostatic complexation of DNA, implying an accumulation of the DNA macromolecules on the surface of the hybrid tricomponent complexes. Magnetophoretic experiments verified that the MNPs maintain their magnetic properties after the complexation initially with the copolymer, and subsequently, within the block polyelectrolyte/MNPs/DNA nanostructures.

## 1. Introduction

Polymeric theranostics that can deliver imaging and therapeutic agents simultaneously are being intensively developed to achieve dual-functionality for the treatment of threatening disorders. A plethora of scientific papers has been published to provide multi-functionality in treatment applications [1,2,3,4,5,6,7,8]. Special attention is given to the design of polymers that are biodegradable and biocompatible, additionally providing solubility and stability to the formed multifunctional hybrid nanostructures.

The early generation of double hydrophilic block copolymers (DHBCs), consisting of two water-soluble blocks of different chemical nature in the macromolecular chain [9], has opened up new avenues of research in the fields of material science, pharmacy, biochemistry and polymer science. The amphiphilicity and self-assembly of DHBCs in aqueous media are driven by changes in the ionic strength, temperature, pH or by complexation with appropriate molecules [10,11,12,13,14,15,16]. A polyelectrolyte block incorporated in DHBCs has the ability to interact/complex with oppositely charged components, such as imaging agents or biomacromolecules, while the neutral hydrophilic blocks promote solubilization in water and provide stabilization of the formed hybrid nanostructures in aqueous dispersions [17,18,19]. The electrostatic interactions between the polyelectrolyte blocks and the oppositely charged compounds govern the complex structure formation [20,21].

Magnetic nanoparticles (MNPs) have been used in a multitude of biomedical applications including magnetic resonance imaging (as negative contrast agents), gene therapy, targeted drug delivery, and magnetic hyperthermia treatment (under alternating magnetic fields) [22,23,24,25,26], rendering them ideal for the design of hybrid nanostructures based on polymers.

The development of hybrid nanostructures formed by DHBCs embedded with hydrophilic MNPs have been reported by the Berret [27] and Held [28] research groups. In addition, Minko et al. reported the formation of hybrid complexes encapsulating MNPs and polyelectrolytes, under the application of a magnetic field [29]. Other research groups have found that the formation of mixed hybrid nanostructures formed by DHBCs and MNPs leads to the effective stabilization of MNPs in aqueous dispersions [17,18,19].

DHBCs conjugated electrostatically with MNPs and biological molecules (deoxyribonucleic acid (DNA) and ribonucleic acid (RNA)) allow for the development of polymeric theranostics, ameliorating both diagnosis (by exploiting the magnetic properties) and therapy of various genetic disorders (by replacing/silencing defective genes). Recent research has demonstrated that polymer-based nanocarriers incorporating MNPs and biomolecules (DNA, plasmid-DNA (pDNA), small-interfering-RNA, (siRNA)) offer a plethora of advantages under the application of a magnetic field [30,31,32,33,34,35]. Several studies have proven that poly(ethylenimine) (PEI) complexed with MNPs for DNA delivery is the most efficient gene carrier for targeted gene therapy under the influence of a magnetic field [36,37,38].

In the present contribution, we developed multifunctional hybrid spherical-shaped nanostructures utilizing a biocompatible DHBC synthesized by Reversible Addition–Fragmentation Chain Transfer (RAFT) polymerization, namely the poly[oligo(ethylene glycol) methacrylate]-*b*-poly[(vinyl benzyl trimethylammonium chloride)] (POEGMA-*b*-PVBTMAC), as the base for electrostatic complexation initially with hydrophilic MNPs, and subsequently, incorporating a short DNA. The electrostatic interactions between the cationic PVBTMAC moieties of the copolymer and the negatively-charged MNPs/DNA govern the formation of polymer/MNPs and polymer/MNPs/DNA hybrid spherical-shaped nanostructures (referred in the text as MNPs–hybrid complexes and magnetopolyplexes, respectively). As a first step, the RAFT-synthesized POEGMA-*b*-PVBTMAC copolymer polyelectrolyte was studied by dynamic and electrophoretic light scattering techniques (DLS, ELS) to assess the size and the surface potential, as well as its solution behavior under the influence of solution ionic strength. Fluorescence spectroscopy (FS) measurements, using pyrene as the fluorescent probe, were also conducted to determine the microenvironment polarity of the copolymer’s environment at different concentrations of salt. Physicochemical studies were expanded also to MNPs-hybrid complexes and magnetopolyplexes using a plethora of physicochemical methods, including DLS and ELS techniques. Magnetophoresis experiments were conducted for the MNPs-hybrid complexes, before the complexation of the short nucleic acid, to investigate their behavior under the application of a magnetic field using ultraviolet spectroscopy (UV-Vis). To confirm that the magnetopolyplexes maintain the magnetic properties of the inorganic part after electrostatic binding with the short nucleic acid, magnetophoresis measurements were repeated. Finally, the structure of both MNPs-hybrid complexes and magnetopolyplexes was determined by cryogenic transmission electron microscopy measurements (cryo-TEM).

## 2. Materials and Methods

### 2.1. Materials

Synthesis of cobalt ferrite nanoparticles (CoFe_2_O_4_ NPs): hexane (Alfa Aesar, 99%, Haverhill, MA, USA), oleic acid (Penta, 97%, Prague, Czech Republic), ethanol absolute (Lach-ner, 99.8%, Neratovice, Czech Republic), 1-pentanol (Lach-ner, 99.8%), 1-octanol (Sigma-Aldrich, 99%, Athens, Greece), sodium hydroxide (Penta, 97%), iron (III) nitrate nonahydrate (Sigma-Aldrich, 98%), cobalt (II) nitrate hexahydrate (Penta, 99%); bovine serum albumin (BSA, 99%, Sigma-Aldrich), sodium chloride (NaCl, 99.5%, Sigma-Aldrich); pyrene was used as the hydrophilic fluorescent probe and received from Sigma-Aldrich. DNA sodium salt (with~113 bp) from salmon testes was received from Sigma-Aldrich. The monomers vinyl benzyl trimethylammonium chloride (VBTMAC) and oligo(ethylene glycol) methacrylate (OEGMA, *n* = 9) were purchased from Sigma-Aldrich.

### 2.2. POEGMA-b-PVBTMAC Block Copolymer Synthesis

**Scheme 1:**
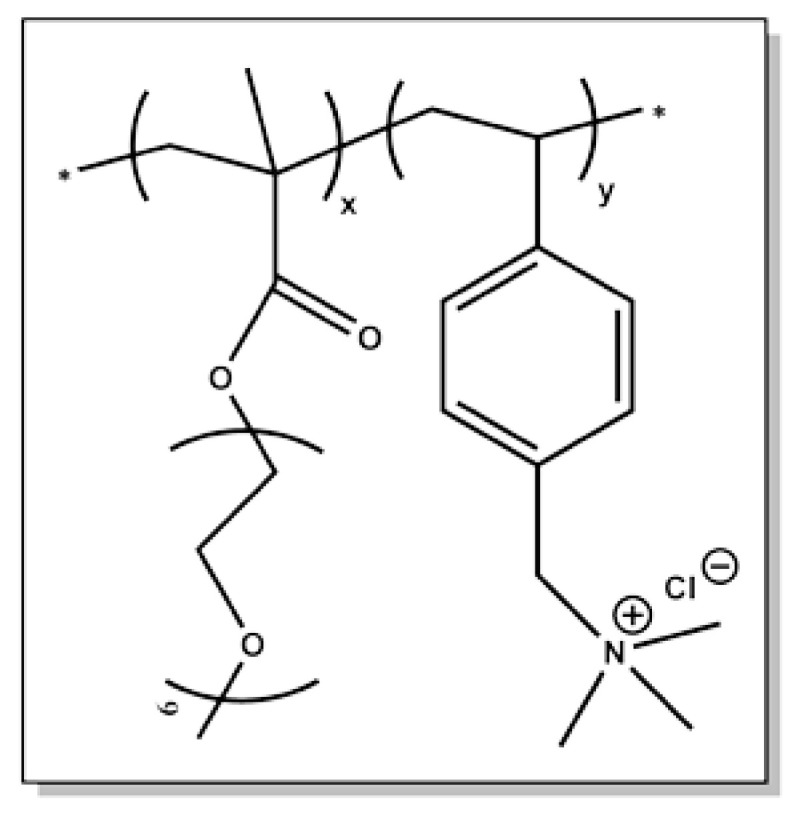
Chemical structure of the POEGMA-*b*-PVBTMAC copolymer synthesized by RAFT polymerization.

The synthesis of PVBMAC homopolymer and POEGMA-*b*-PVBTMAC DHBC was performed by the RAFT polymerization technique in aqueous media. The synthetic procedure has been described in detail in a previous work [39]. The chemical structure and molecular characteristics of the copolymer are presented in Scheme 1 and Table 1, respectively.

### 2.3. Synthesis of CoFe_2_O_4_ NPs

Hydrophobic CoFe_2_O_4_ nanoparticle dispersions were synthetized from corresponding iron and cobalt oleates under hydrothermal conditions (see details in previous work). Briefly, the metal oleates were prepared from iron and cobalt nitrates and sodium oleate solutions. These were mixed and refluxed with hexane, while water phase was discarded after. Hexane solvent was replaced by 1-pentanol and desired amount of this mixture was placed into a Teflon lined steel autoclave (Berghof), together with 1-octanol and distilled water. Hydrothermal treatment took place in a preheated furnace at 180 °C for 10 h. Such a technique gave hydrophobic nanoparticle dispersion. The particle surface was covered by oleic acid, which was later easily replaced by citric acid to convert hydrophilic particles [40].

### 2.4. Self-Assembly of POEGMA-b-PVBTMAC Block Copolymer

Due to the polyelectrolyte nature of the POEGMA-*b*-PVBTMAC DHBC, its solution behavior was studied in aqueous solutions using two dissolution protocols. The first protocol includes the direct copolymer dissolution in water for injection at two concentrations of 5 × 10^−4^ g/mL and 10^−3^ g/mL. The second protocol concerns the direct copolymer dissolution in NaCl solutions of different concentrations (0.01, 0.15, 0.3 and 0.5 M) at a polymer concentration of 5 × 10^−4^ g/mL. All copolymer solutions were filtered through 0.45 μm pore size filters and allowed to stand overnight for equilibration before measurements.

### 2.5. Preparation of MNPs–Hybrid Complexes

The double-hydrophilic POEGMA-*b*-PVBTMAC copolymer was directly dissolved in water for injection at a concentration of 5 × 10^−4^ g/mL. The hybrid nanostructures were formed by vigorously mixing the copolymer solution with an aqueous dispersion of CoFe_2_O_4_ NPs to prepare the mixture with the desired wt. % concentration of the CoFe_2_O_4_ NPs. Particularly, an aqueous dispersion of 0.5 mg of the CoFe_2_O_4_ NPs was added to the copolymer solution to prepare a 20 wt. % concentration of the CoFe_2_O_4_ NPs in the mixture. The POEGMA-*b*-PVBTMAC + 0.5 mg CoFe_2_O_4_ complexes were allowed to stand for ten minutes under gentle stirring and a 1:10 dilution of the formed hybrid complexes was prepared and left for overnight equilibration. High concentrations of CoFe_2_O_4_ NPs (40 and 80 wt. %) produced hybrid complexes with poor stability in aqueous media over time.

### 2.6. Preparation of Magnetopolyplexes

The magnetopolyplexes were formed by mixing gently the POEGMA-*b*-PVBTMAC + 0.5 mg CoFe_2_O_4_ complexes with DNA stock solutions in the desired *N/P* ratios. The polyplexes were prepared at *N/P* ratios in the 1 to 7 range by keeping the concentration of the hybrid complexes constant and varying that of DNA, at ambient temperature under stirring. The POEGMA-*b*-PVBTMAC copolymer was directly dissolved in 0.01 M NaCl at a concentration of 5 × 10^−4^ g/mL. An aqueous dispersion of 0.5 mg of the CoFe_2_O_4_ NPs was added to the copolymer solution to prepare a 20 wt. % concentration of the CoFe_2_O_4_ NPs in the mixture. The DNA stock solution of 0.01 M NaCl was prepared at 2 × 10^−4^ g/mL for the subsequent mixing and formation of the POEGMA-*b*-PVBTMAC + 0.5 mg CoFe_2_O_4_ + DNA polyplexes. Indicatively, 2 mL of the POEGMA-*b*-PVBTMAC + 0.5 mg CoFe_2_O_4_ solution was added to each *N/P*ratio before mixing with the appropriate volume of DNA solution. The magnetopolyplexes were allowed to stand for ten minutes under gentle stirring and left for subsequent equilibration overnight.

### 2.7. Methods

DLS measurements were carried out on an ALV/CGS-3 Compact Goniometer System (ALV GmbH, Siemensstraße 4, 63225 Langen (Hessen), Germany) equipped with an ALV-5000/EPP multi-tau digital correlator with 288 channels and an ALV/LSE-5003 light scattering electronics unit for stepper motor drive and limit switch control. A JDS Uniphase 22-mW He-Ne laser (632.8 nm) was used as the light source. The size data and figures shown in the manuscript are from measurements at 90°. The solutions were filtered through 0.45 μm hydrophilic PTFE filters (Millex-LCR from Millipore, Billerica, MA, USA) before measurements. Toluene was used as the calibration standard. The obtained correlation functions were analyzed by the cumulants method and CONTIN software.

ELS measurements were conducted using a Nano Zeta Sizer (Malvern Instruments Ltd., Malvern, UK) equipped with a 4-mW solid-state laser, operating at 633 nm and a fixed backscattering angle of 173°. The reported zeta-potential values are the average values of 50 runs, using the Henry correction of Smoluchowski equation after equilibration at 25 °C.

FS experiments were performed in order to determine the microenvironment polarity of block copolymer-based solutions at different concentrations of NaCl. Fluorescence spectra were recorded using a Fluorolog-3 Jobin Yvon-Spex as the spectrofluorometer (modelGL3-21). The excitation wavelength used for the measurements was 335 nm and emission spectra were in the range of 355–630 nm. A stock solution of 1 mM pyrene (fluorescent probe) in acetone was prepared and added in the solutions in a ratio of 1 μL per 1 mL polymer solution. Measurements were conducted after evaporation of acetone overnight at room temperature.

UV-Vis absorption spectra of magnetopolyplexes were recorded between 200 and 500 nm wavelength using a Perkin Elmer (Lambda 19) UV-Vis-NIR spectrophotometer (Waltham, MA, USA). The magnetopolyplexes were prepared at *N/P*ratios in the 1 to 7 range and were diluted to get an absorbance value of less than 1. The magnetophoretic experiments were recorded using a Perkin Elmer (Lambda 19) UV-Vis-NIR spectrophotometer (Waltham, MA, USA) by placing a cylindrical Nd-Fe-B magnet (dimensions: diameter = 20 mm, thickness = 10 mm, magnetization unit: N45, attraction/repulsion strength: max 16 kg) next to the cuvette holder. The wavelength for the measurements was set at 450 nm and the absorbance of the solutions containing the MNPs–hybrid complexes, as well as the magnetopolyplexes, was measured for 1 h under the influence of the magnet. It should be emphasized that the absorption at 450 nm is related to the presence of MNPs and not of the copolymer.

Cryo-TEM images were obtained using a Tecnai F20 X TWIN microscope (FEI Company, Hillsboro, OR, USA) equipped with a field emission gun, operating at an acceleration voltage of 200 kV. Images were recorded on the Gatan Rio 16 CMOS 4 k camera (Gatan Inc., Pleasanton, CA, USA) and processed with Gatan Microscopy Suite (GMS) software (Gatan Inc., Pleasanton, CA, USA). Specimen preparation was done by vitrification of the aqueous solutions on grids with holey carbon film (Quantifoil R 2/2; Quantifoil Micro Tools GmbH, Großlöbichau, Germany). Prior to use, the grids were activated for 15 s in oxygen plasma using a Femto plasma cleaner (Diener Electronic, Ebhausen, Germany). Cryo-samples were prepared by applying a droplet (3 μL) of the suspension to the grid, blotting with filter paper and immediate freezing in liquid ethane using a fully automated blotting device Vitrobot Mark IV (Thermo Fisher Scientific, Waltham, MA, USA). After preparation, the vitrified specimens were kept under liquid nitrogen until they were inserted into a cryo-TEM-holder Gatan 626 (Gatan Inc., Pleasanton, CA, USA) and analyzed in the TEM at −178 °C.

The X-ray diffraction (XRD) profiles were measured on a Bruker D8 Advance Twin diffractometer (Billerica, MA, USA) with a Cu tube (*λ* = 1.5418^°^A) and LYNXEYE_XE_T 1D detector. X-rays were generated under 40 kV and 40 mA tube operating condition. Scans were over the range of 20°–70° 2*θ* with step size 0.1° and scan speed 5 s/step.

## 3. Results and Discussion

### 3.1. Synthesis of POEGMA-b-PVBTMAC Diblock Copolymer

The double hydrophilic POEGMA-*b*-PVBTMAC copolymer was synthesized by RAFT polymerization and the molecular characterization is presented and discussed in a previous work from our laboratory [39]. The molecular characteristics are shown in Table 1 and the chemical structure of the copolymer is presented in Scheme 1.

### 3.2. Characterization of CoFe_2_O_4_ NPs

DLS and XRD measurements implemented to assess the size and the crystalline structure of the CoFe_2_O_4_ NPs are presented in the Supplementary Material (SM) in Figures S1 and S2 [41]. ELS measurements were also carried out for the determination of the zeta-potential (*ζ_pot_*) value that is reported in SM, Table S1. The MNPs carry a negative charge as a result of the synthesis procedure. Hysteresis loops are also exhibited in SM, Figure S3, to clearly demonstrate the magnetic property of CoFe_2_O_4_ NPs, where the saturation magnetization (M_S_) and coercive field (μ_0_H_C_) values are listed in SM, Table S2.

### 3.3. Physicochemical Characterization of the POEGMA-b-PVBTMAC Copolymer

**Figure 1:**
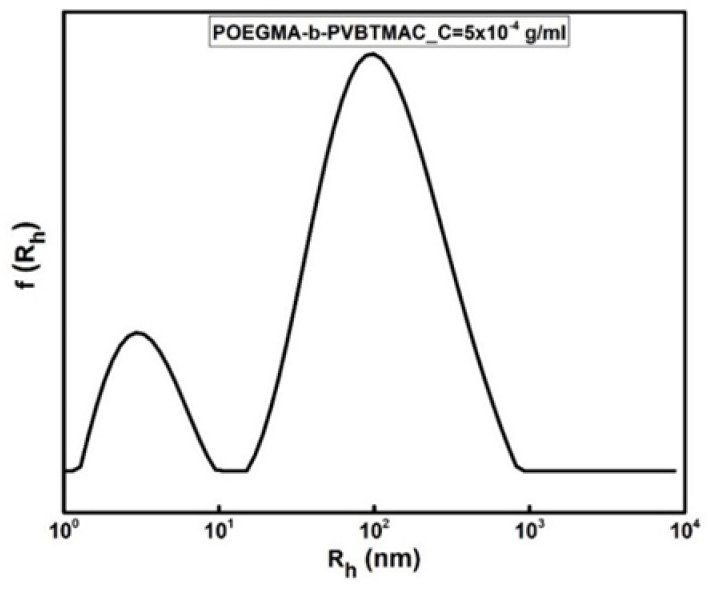
Size distribution from Contin for the POEGMA-*b*-PVBTMAC diblock at 90° in aqueous solutions (solvent: water for injection).

The physicochemical properties of the polyelectrolyte block copolymer in aqueous media were determined by FS, DLS and ELS measurements. The self-assembly of the POEGMA-*b*-PVBTMAC copolymer was studied with two types of media, water for injection and NaCl aqueous solutions, and at different solution concentrations (5 × 10^−4^ g/mL, 10^−3^ g/mL). DHBCs are expected to exist as single molecules in a random coil conformation in aqueous solutions unless triggered with an appropriate chemical, physical or biochemical stimulus [42]. However, the POEGMA-*b*-PVBTMAC nanostructures coexist as singles chains and aggregates in water, exhibiting a bimodal size distribution in DLS, as the PVBTMAC block contains hydrophobic domains stabilized by the hydrophilic POEGMA segments, resulting in polymer association. The hydrodynamic radius (R_h_) and size polydispersity index (PDI) values of the POEGMA-*b*-PVBTMAC nanostructures formed in aqueous media are presented in Table 2 at C = 5 × 10^−4^ g/mL, pH = 7 and 25 °C. The intensity weighted size distribution plot from Contin analysis in Figure 1 reveals the existence of two peaks that correspond to copolymer unimers (*R_h_* = 3 nm) and aggregates (*R_h_* = 109 nm). The size distribution and the DLS characteristics of the copolymer at C = 10^−3^ g/mL in aqueous solutions are presented in SM, in Figure S4 and Table S3.

The surface potential of the POEGMA-*b*-PVBTMAC copolymer was determined by ELS measurements in aqueous media and appears strongly positive, *ζ_pot_* = +20 mV (Table 2), due to the permanently charged cationic PVBTMAC moieties of the copolymer.

**Figure 2:**
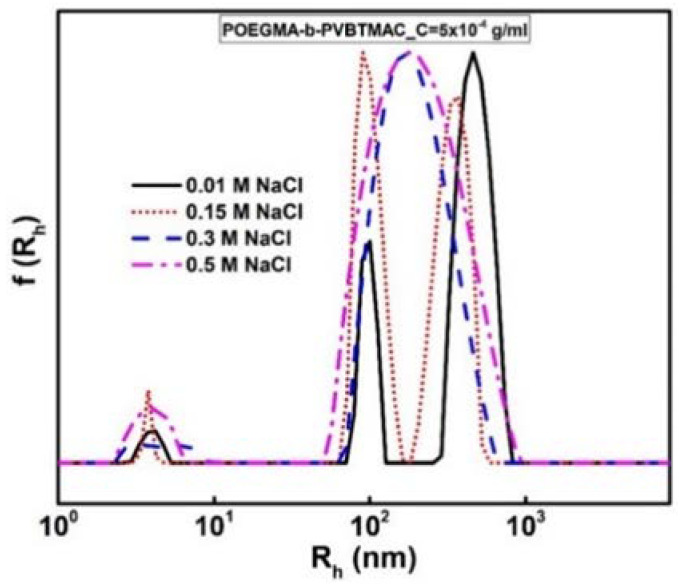
Comparison of the size distributions from Contin for the POEGMA-*b*-PVBTMAC diblock at different concentrations of NaCl at 90°.

DLS measurements for the POEGMA-*b*-PVBTMAC copolymer were also conducted using NaCl solutions of different salt concentrations (0.01, 0.15, 0.3 and 0.5 M) since PVBTMAC is a cationic polyelectrolyte block and its state in aqueous media is expected to be influenced by the presence of low molecular weight salt. The measurements were performed at 5 × 10^−4^ g/mL, pH = 7 and 25 °C. A comparison of size distributions of the POEGMA-*b*-PVBTMAC copolymer in NaCl solutions with different concentrations is presented in Figure 2 at 90°. The size distribution plot at 0.01 M NaCl reveals three peaks, implying the presence of block copolymer unimers at 4 nm, copolymer aggregates at 97 nm and larger aggregates (supra-aggregates) at 469 nm (Table 3).

In the size distribution plot of 0.15 M NaCl (the physiological ionic strength), three intensity peaks appear, similarly-sized to the intensity peaks formed at 0.01 M NaCl. The third population represents aggregates of 337 nm (decreased size and lower intensity peak compared to the 0.01 NaCl case). Noticeably, the size distributions plots of 0.3 and 0.5 M NaCl, display two discrete populations at 4 and 190 nm, revealing copolymer unimers, as well as copolymer aggregates. The intensity peaks at 190 nm emerge between the peaks at 97 and 469 nm, which are observed in the size distribution plot of 0.01 M NaCl, a fact that probably indicates the incorporation of these two peaks into one quite broader peak as salt concentration increases. Conclusively, the POEGMA-*b*-PVBTMAC copolymer self-assembly in NaCl solutions shows variations in the number and size of aggregate populations (Figure 2), in contrast to the copolymer dissolution in aqueous media (Figure 1). This may be a consequence of polyelectrolyte phenomena (screening effects) as well as hydrophobic interactions, originating from the hydrophobic backbone and phenyl side groups of PVBTMAC block.

ELS measurements were carried out at 0.01, 0.15, 0.3 and 0.5 M NaCl for the determination of the surface potential of the copolymer aggregates. The presence of salt in the polyelectrolyte POEGMA-*b*-PVBTMAC copolymer evidenced a strong impact on the surface potential, leading to slightly negative ζ_pot_ values with small differences between them: −1 mV at 0.01 M NaCl, −3 mV at 0.15 M NaCl and −4 mV at 0.5 M NaCl.

**Figure 3:**
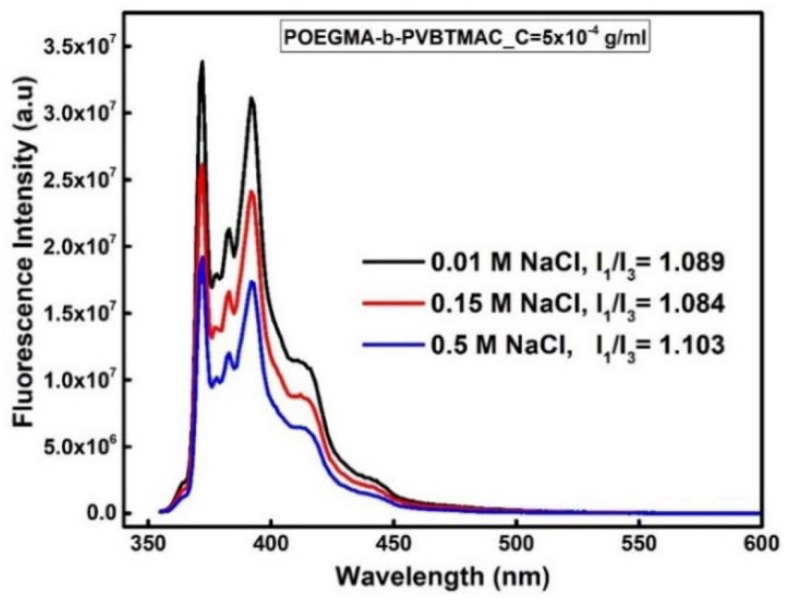
Fluorescence pyrene emission spectra from POEGMA-*b*-PVBTMAC solutions at different NaCl concentrations.

The microenvironment polarity of the block polyelectrolyte solutions was studied at different concentrations of NaCl (0.01, 0.15 and 0.5 M) using the FS technique and pyrene as the fluorescent probe. A comparison of the fluorescence spectra for the POEGMA-*b*-PVBTMAC copolymer at different concentrations of NaCl, is presented in Figure 3. The double hydrophilic POEGMA-*b*-PVBTMAC copolymer possesses a water soluble PVBTMAC segment which contains a benzyl group that offers a hydrophobic domain, rendering the copolymer ideal for a number of demanding applications. Calculated relative intensity ratios, I_1_/I_3_ (ca. 1.1, Figure 3), confirm a nonpolar microenvironment that slightly changes with the addition of NaCl.

### 3.4. Effect of Solution Ionic Strength on the Polyelectrolyte Copolymer Behavior

**Figure 4:**
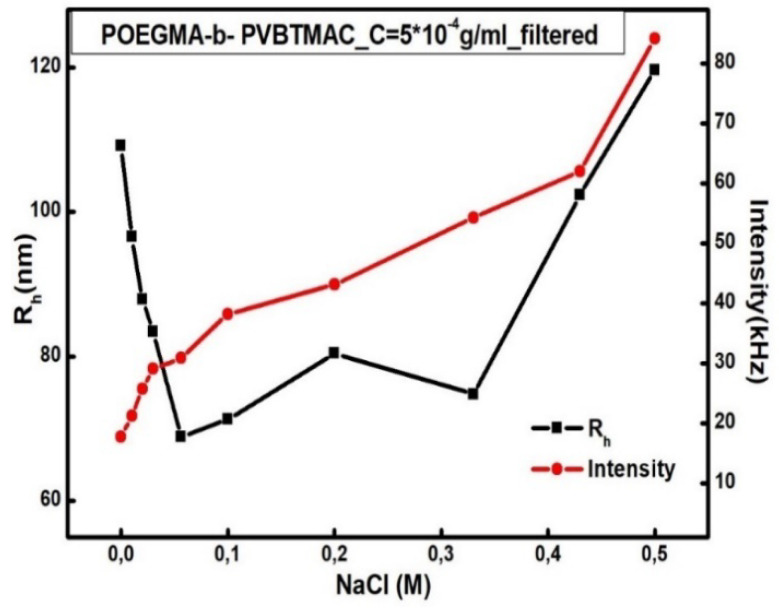
R_h_ and scattered intensity as a function of ionic strength ([NaCl]) for POEGMA-*b*-PVBTMAC at C = 5 × 10^−4^ g/mL.

The addition of NaCl in polyelectrolyte copolymer solutions usually plays a central role in polymer’s solubility and size. The effect of ionic strength on the POEGMA-*b*-PVBTMAC diblock is rooted in the strong polyelectrolyte nature of the PVBTMAC block. The DLS measurements of the POEGMA-*b*-PVBTMAC copolymer were conducted at two copolymer concentrations of 5 × 10^−4^ g/mL and at 10^−3^ g/mL (data from the latter being provided in SM, in Figure S4). In Figure 4, the dependence of R_h_ and scattered intensity on the NaCl concentration is depicted for the POEGMA-*b*-PVBTMAC copolymer. Regarding Figure 4, a decrease in R_h_ of **≈** 40 nm is observed as the concentration of NaCl rises from 0 to 0.056 M, followed by an increase in R_h_ until the concentration of NaCl reaches 0.5 M. The parallel increase in the scattered intensity indicates the formation of aggregates of increasing mass upon the addition of salt. The charge screening from NaCl addition, some hydrophobic interactions emanating from the benzyl group of PVBTMAC, along with the enhanced system’s hydrophilicity (coming from POEGMA segment and the incorporation of water molecules), yield some important metastable aggregation states.

A completely different polyelectrolyte copolymer behavior is presented in SM, in Figure S5 at C = 10^−3^ g/mL, uncovering the formation of aggregates resistant to salt screening effects.

### 3.5. Electrostatic Complexation of POEGMA-b-PVBTMAC Copolymer with MNPs

**Figure 5:**
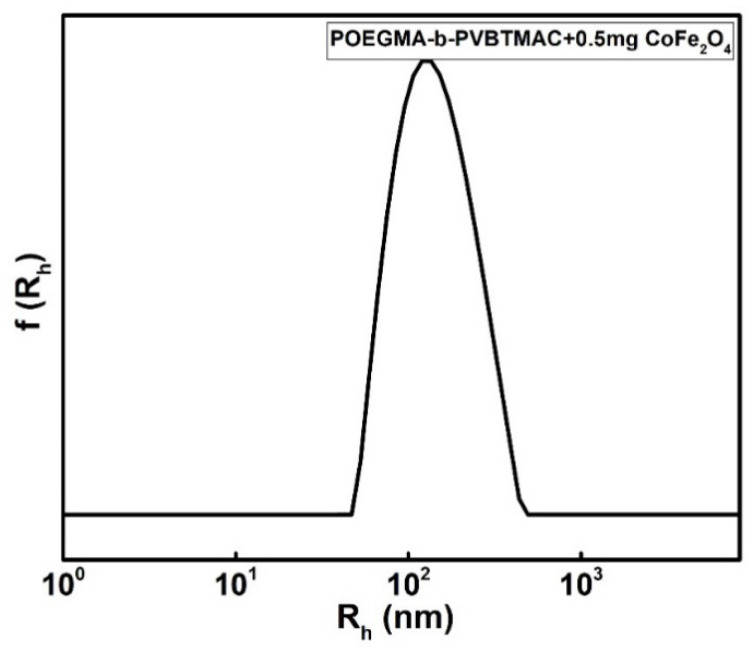
Size distribution from Contin for POEGMA-*b*-PVBTMAC/MNPs hybrid complexes at C = 5 × 10^−4^ g/mL in aqueous solutions and 90°.

Electrostatic interactions between the polycationic moieties of PVBTMAC and the negatively charged MNPs, are the driving force for the incorporation of the hydrophilic CoFe_2_O_4_ NPs in the hybrid complexes. DLS and ELS measurements were performed for the POEGMA-*b*-PVBTMAC + 0.5 mg CoFe_2_O_4_ complexes at a concentration of 5 × 10^−4^ g/mL in aqueous solutions. Figure 5 exhibits a monomodal symmetrical and narrow size distribution from Contin analysis, indicating the participation of all chains in the formation of the hybrid complexes after complexation with the MNPs. The R_h_, PDI and ζ_pot_ values of the formed complexes are provided in Table 2. DLS results indicate that the hydrophilic MNPs participate effectively in the co-assembly process, as the size and mass of the hybrid nanostructures are significantly increased after the complexation with MNPs. Additionally, the PDI value became quite small after complexing CoFe_2_O_4_ NPs to the copolymer, reflecting a uniform size distribution. The obtained results are a consequence of a large complexation tendency between the components and a strongly synergistic co-assembly. Having in mind that DLS is a low resolution technique and gives intensity-weighted populations, provided data from DLS (larger particle sizes/uniform morphology) are not expected to be fully consistent with cryo-TEM results shown in Figure 10 (which is a number-weighted and contrast-dependent technique) and presented two types of hybrid and spherical-shaped complexes.

ELS measurements detected a strong positive *ζ_pot_* = +14 mV of the MNPs–hybrid complexes due to the presence of the relatively large and permanently cationic PVBTMAC moieties decorating them, despite the existence of the negatively charged MNPs.

### 3.6. Interaction of BSA with the Diblock/MNPs Hybrid Complexes

An investigation of the interaction between the POEGMA-*b*-PVBTMAC/MNPs hybrid complexes with bovine serum albumin (BSA) is presented in the SM in order to provide a better understanding of its potential applications in biological environments. The dependence of R_h_ and scattered intensity on BSA concentration is depicted in SM, Figure S6, revealing the formation of hybrid complexes having lower mass upon gradual addition of BSA. There is definitely an interaction between BSA and hybrid complexes which apparently leads to partial dissociation of initially formed diblock/MNPs complexes. Obviously, upon interaction with BSA, diblock/MNPs/BSA complexes with looser structure are formed at high protein concentrations, as the observed increase in R_h_ indicates. Presumably, the presence of hydrophilic POEGMA chains does not allow the formation of very large aggregates in the presence of BSA.

### 3.7. Electrostatic Complexation of POEGMA-b-PVBTMAC Copolymer with MNPs and DNA

The complexation of the POEGMA-*b*-PVBTMAC + 0.5 mg CoFe_2_O_4_ with DNA was achieved through electrostatic coupling of the positively charged amino groups of PVBTMAC and the negatively charged phosphate groups of DNA. The ability of PVBTMAC-based copolymers to form polyplexes with DNA molecules has been examined in an earlier study [39]. According to a recent study, the PVBTMAC-based polymers are promising candidates for siRNA delivery [43]. Ternary polyplexes containing POEGMA-*b*-PVBTMAC (co)polymer, DNA and gold NPs were formed with success recently [44].

**Figure 6:**
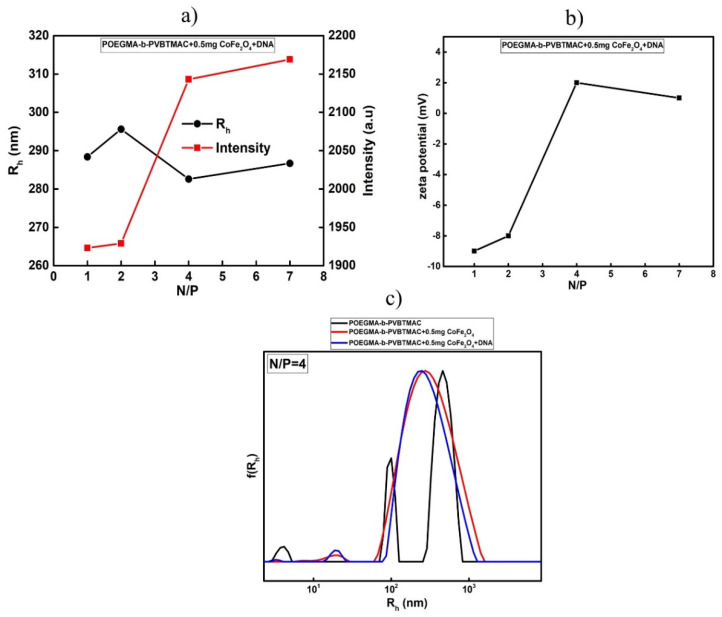
(**a**) *R_h_*, scattered intensity and (**b**) *ζ* potential as a function of *N/P* ratio for the formed magnetopolyplexes at *N*/*P* = 7, 4, 2 and 1, (**c**) comparison of the size distributions from CONTIN analysis for POEGMA-*b*-PVBTMAC copolymer, MNPs–hybrid complexes and magnetopolyplexes at *N*/*P* = 4 and at 0.01 M NaCl.

The POEGMA-*b*-PVBTMAC + 0.5 mg CoFe_2_O_4_ + DNA magnetopolyplexes were prepared at different *N/P*ratios with a short DNA (≈113 bp). Figure 6 summarizes the DLS and ELS experiments carried out for the magnetopolyplexes. DLS data in Figure 6a reveal small deviations in R_h_ values and an increase in scattered intensity as the *N/P*ratio rises from 1 to 7. Specifically, the formation of higher mass magnetopolyplexes with relatively constant dimensions is observed as POEGMA-*b*-PVBTMAC + 0.5 mg CoFe_2_O_4_ complexes prevail at high *N/P* ratios.

Negative ζ_pot_ values of the magnetopolyplexes, as determined by ELS measurements, are presented in Figure 6b at low *N/P* ratios (1 and 2), where DNA is in excess and more non-complexed phosphate groups exist in the solution. On the other hand, positive ζ_pot_ values appear at high *N/P* ratios (4 and 7) where positively charged tertiary amino groups of the copolymer prevail. A comparison of the size distributions for POEGMA-*b*-PVBTMAC copolymer, POEGMA-*b*-PVBTMAC + 0.5 mg CoFe_2_O_4_ complexes and POEGMA-*b*-PVBTMAC + 0.5 mg CoFe_2_O_4_ + DNA magnetopolyplexes at *N*/*P* = 4 is presented in Figure 6c at 90°. The size distribution of the copolymer in Figure 6 (black line) (presented also in Figure 2) shows three peaks at 0.01 M NaCl. After the complexation with CoFe_2_O_4_ NPs, the size distribution becomes almost monomodal (Figure 6c—red line), uncovering higher mass hybrid complexes of 300 nm at 0.01 M NaCl, leading to the conclusion that the presence of the MNPs play a fundamental role in the co-assembly in NaCl solutions. The electrostatic complexation of nucleic acids generated higher mass magnetopolyplexes of 283 nm at *N/P* ratio = 4 as seen in Figure 6c (blue line), with smaller PDI value than that of MNPs–hybrid complexes. All DLS results before and after the complexation with DNA are summarized in Table 3.

Furthermore, UV–Vis measurements were performed to further characterize the complexation of the POEGMA-*b*-PVBTMAC + 0.5 mg CoFe_2_O_4_ with DNA molecules [45,46]. The results are provided in SM, in Figure S7.

### 3.8. Behavior of Magnetopolyplexes in the Presence of Salt

**Figure 7:**
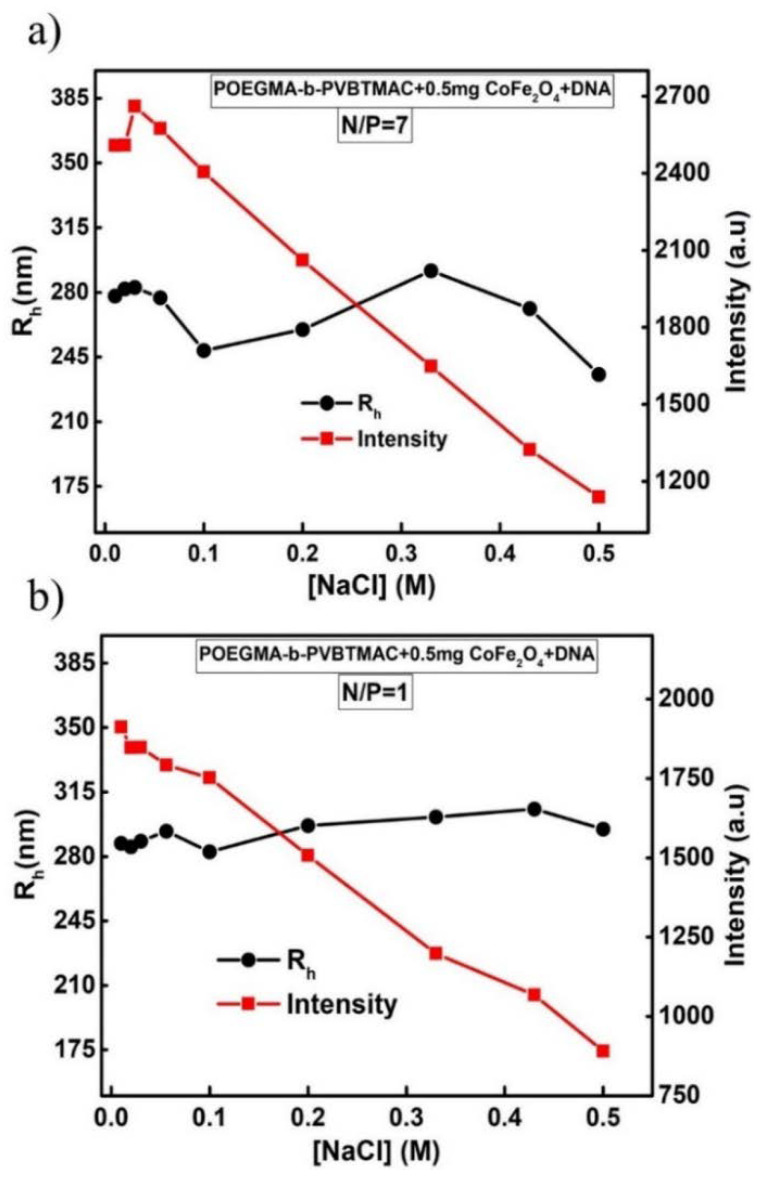
*R_h_* and scattered intensity as a function of ionic strength ([NaCl]) for magnetopolyplexes at (**a**) *N*/*P* = 7 and (**b**) *N*/*P* = 1.

The solutions of POEGMA-*b*-PVBTMAC + 0.5 mg CoFe_2_O_4_ + DNA polyplexes that exhibited the greater stability over time were further studied under the influence of ionic strength through DLS measurements. Figure 7 displays the *R_h_* and scattered intensity values as a function of ionic strength for the selected polyplex solutions at (a) *N*/*P* = 7 and (b) *N*/*P* = 1. At both *N/P* ratios, small differences in R_h_ of magnetopolyplexes are apparent, while intensity initially remains relatively constant, for NaCl concentration up to 0.056 M, and then, at 0.1 M, NaCl starts gradually to decrease as ionic strength increases. Although magnetopolyplexes exist even at the highest ionic strength utilized, the weaker electrostatic interactions between DNA and MNPs–hybrid complexes result in magnetopolyplexes of lower mass that are partially disintegrated upon the addition of NaCl, due to electrostatic screening.

### 3.9. Magnetophoretic Experiments for Diblock/MNPs Hybrid Complexes and Magnetopolyplexes

**Figure 8:**
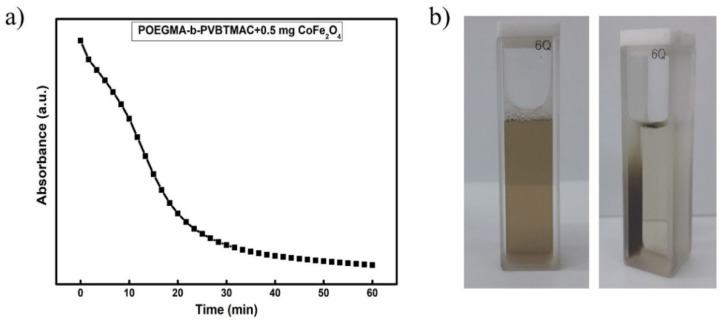
(**a**) Magnetophoresis plot showing absorbance as a function of time for MNPs-hybrid complexes (*λ* = 450 nm), (**b**) MNPs-hybrid complexes before and after the application of external magnetic field (using a magnet) for the magnetophoresis experiments.

Magnetophoretic experiments for the POEGMA-*b*-PVBTMAC + 0.5 mg CoFe_2_O_4_ complexes were performed to determine the magnetic properties of the spherical-shaped hybrid nanostructures after complexation of the components. The magnetophoresis plot in Figure 8a depicts the absorbance as a function of time for the POEGMA-*b*-PVBTMAC + 0.5 mg CoFe_2_O_4_ complexes.

The critical decrease in absorbance at 450 nm is associated with the strong response of the MNPs–hybrid complexes under the influence of the external magnetic field. According to Figure 8b, the MNPs–hybrid complexes are gathered in the side of the measuring cell where the magnet is placed, revealing a significant decline in the absorbance of the solution within the first 20 min, followed by a gradual and smaller decrease until the end of the measurement, as depicted in Figure 8a.

**Figure 9:**
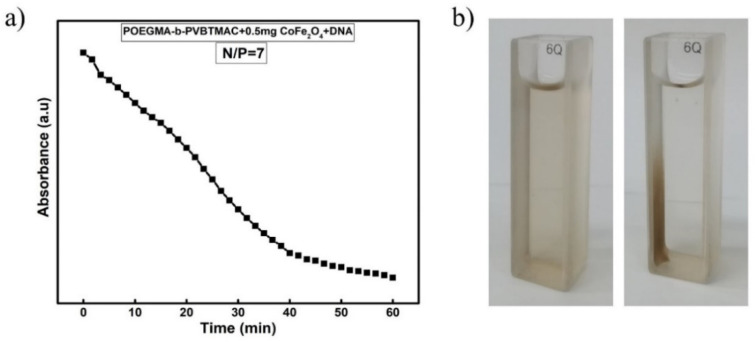
(**a**) Magnetophoresis plot showing absorbance as a function of time for magnetopolyplexes (*λ* = 450 nm) at *N*/*P* = 7, (**b**) magnetopolyplexes before and after the application of magnetic field (using a magnet) for the magnetophoresis experiments.

The magnetic response of the magnetopolyplexes was also determined by magnetophoretic measurements under the application of an external magnetic field. The magnetophoresis graph in Figure 9a illustrates the time dependence of the absorbance for the POEGMA-*b*-PVBTMAC + 0.5 mg CoFe_2_O_4_ + DNA magnetopolyplexes at *N*/*P* = 7, while Figure 9b presents the accumulation of CoFe_2_O_4_ NPs in the side of the measuring cell before and after the application of magnetic field. The obtained UV-Vis results are in the same line with those of the MNPs-hybrid complexes, indicating that DNA electrostatic binding does not influence the magnetic properties of the magnetopolyplexes, which remain responsive to magnetic fields.

### 3.10. Cryo-TEM of the MNPs-Hybrid Complexes and DNA-Hybrid Polyplexes

**Figure 10:**
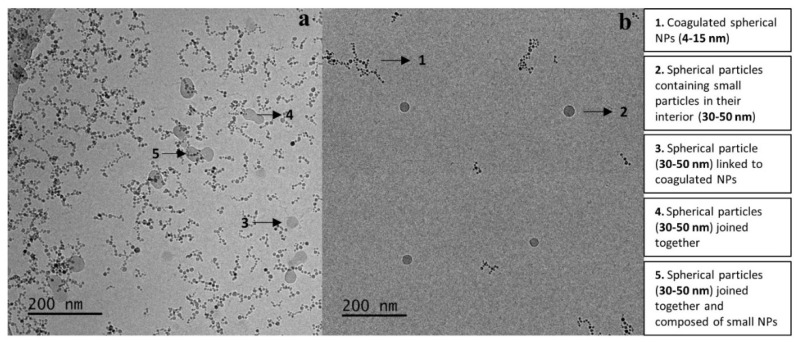
Cryo-TEM micrographs (**a**,**b**) of the MNPs-hybrid complexes and explanations of the structures observed at different points of the images.

**Figure 11:**
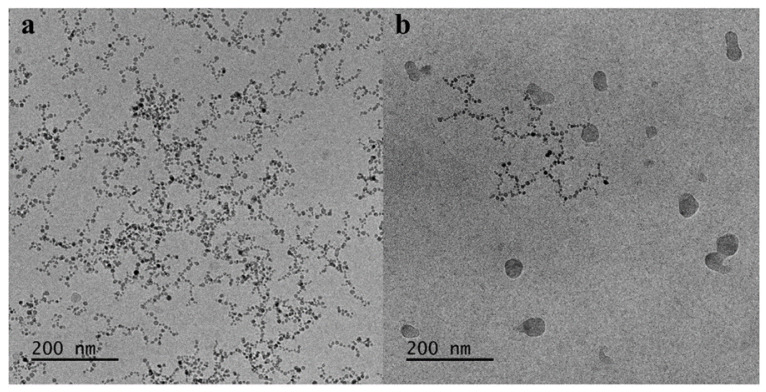
Cryo-TEM micrographs (**a**,**b**) of the magnetopolyplexes at *N*/*P* = 7.

**Figure 12:**
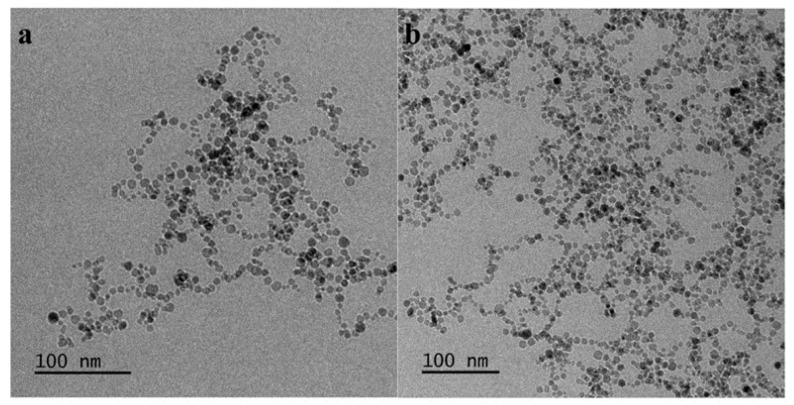
Cryo-TEM micrographs (**a**,**b**) of the magnetopolyplexes at *N*/*P* = 4.

Cryo-TEM experiments provided a closer morphological description of the CoFe_2_O_4_ NPs, firstly, after their complexation with POEGMA-*b*-PVBTMAC copolymer (Figure 10), and secondly, with short DNA (Figure 11 and Figure 12). The measurements were conducted at a concentration of 5 × 10^−4^ g/mL. The electrostatic complexation of the copolymer with the hydrophilic MNPs generated spherically-shaped structures (noted with black arrows in Figure 10). The domains of higher electron density and strong contrast in the hybrid systems are mainly due to the inorganic NPs, while the polymeric material is barely visible in the micrographs at the outer part of the structures. Two kind of NPs emerged based on their size: (a) spherical NPs with diameter 4–15 nm and (b) spherical NPs with diameter 30–50 nm. In particular, small-sized and coagulated NPs of diameter 4–15 nm, are revealed at neutral pH (point 1 in Figure 10b). Larger and well-defined spherical particles with diameter from 30 up to 50 nm, containing other small particles in their interior, are also observed in Figure 10b (point 2). A representative example that probably manifests the copolymer binding with MNPs is presented in Figure 10a (point 3). A spherical particle with smooth surface is linked to the coagulated NPs though copolymer chains which, due to their low contrast, are barely distinguishable. Furthermore, spherical particles that are joined together appear in Figure 10a (point 4) (30–50 nm), while nearly spherical particles bonded together and comprising NPs (30–50 nm) are observed in Figure 10a (point 5). Additional Cryo-TEM micrographs of the POEGMA-*b*-PVBTMAC + 1 mg CoFe_2_O_4_ complexes are provided and discussed in SM, in Figure S8.

Cryo-TEM experiments were also conducted for POEGMA-*b*-PVBTMAC + 0.5 mg CoFe_2_O_4_ + DNA magnetopolyplexes at *N*/*P* = 7 (Figure 11) and *N*/*P* = 4 (Figure 12). It must be noted that the particles retain their spherical shape after the electrostatic complexation with DNA in both ratios. Cryo-TEM measurements on the magnetopolyplexes show undoubtedly an aggregation of the MNPs at *N*/*P* = 7, where two types of CoFe_2_O_4_ NPs arise (Figure 11). Spherical shape particles (4–15 nm) that are either coagulated or joined together in bigger structures (30–50 nm) are formed. Most likely, the aggregation tendency of the cobalt ferrite NPs at *N*/*P* = 7 indicates the accommodation of the DNA macromolecules on the surface of the hybrid diblock/MNPs complexes. Figure 12 confirms this scenario, as areas with larger aggregation of the MNPs unveiled at *N*/*P* = 4 where DNA is in excess. The MNPs in this case, appeared spherical and coagulated (sizes 4–15 nm).

## 4. Conclusions

Multifunctional hybrid spherical-shaped nanostructures formed by a POEGMA-*b*-PVBTMAC DHBC synthesized by RAFT, hydrophilic MNPs and short linear DNA, were developed and studied by exploiting the electrostatic interactions between the cationic PVBTMAC moieties of the copolymer and the negatively charged MNPs and DNA molecules. A detailed physicochemical characterization of the POEGMA-*b*-PVBTMAC copolymer, MNPs-hybrid complexes and magnetopolyplexes, using DLS, ELS, FS, UV-Vis and cryo-TEM techniques, has been provided.

The solution concentration, the solvent media and the gradual addition of salt, cast a strong influence on the self-assembly of the polyelectrolyte copolymer and the co-assembly of the components in MNPs-hybrid complexes. DLS results revealed that POEGMA-*b*-PVBTMAC copolymer self-assembled into aggregates of approximately 110 nm in aqueous solutions, while extra aggregate populations of higher dimensions emerged at 0.01 M NaCl.

The MNPs participated in the co-assembly of the mixed aggregates in both solvent media, leading to almost monomodal size distributions, well-defined diblock/MNPs hybrid complexes and magnetopolyplexes. The positively charged diblock/MNPs hybrid complexes formed aggregates of 139 nm in aqueous solutions and 300 nm in NaCl solutions, as determined by ELS and DLS measurements, showing the influence of solution ionic strength on the size of the nanostructures.

The electrostatic complexation of the diblock/MNPs hybrid complexes with short DNA generated magnetopolyplexes of *R_h_* = 283 nm (*N*/*P* = 4) with narrower size distribution than MNPs-hybrid complexes at 0.01 M NaCl. Mass, size, surface potential and the ability of the MNPs-hybrid complexes to interact with DNA molecules, strongly depend on the *N/P*ratios, as indicated by DLS and ELS techniques. Lower mass magnetopolyplexes with relatively constant *R_h_* values are produced upon adding salt.

Cryo-TEM measurements uncovered differences in the morphology between the MNPs-hybrid complexes and the resulting magnetopolyplexes, indicating a significant aggregation tendency of the MNPs upon electrostatic complexation with DNA, that may be attributed to accumulation of the DNA macromolecules on the surface of the hybrid diblock/MNPs complexes. The CoFe_2_O_4_ NPs maintain their magnetic properties after the complexation with the copolymer and nucleic acids, as proved by the magnetophoretic experiments, enriching the hybrid ternary complexes with magnetic field responsiveness. The physicochemical results of the present hybrid spherical-shaped nanostructures pave the way for further biopharmaceutical evaluation, possibly upgrading them to effective and multifunctional non-viral vectors and theranostic nanoparticles in gene therapy and bioimaging.

## Figures and Tables

**Table 1:** Molecular characteristics of the PVBTMAC homopolymer and POEGMA-*b*-PVBTMAC copolymer.

Sample	Mn PVBTMAC	Mn POEGMA	% PVBTMAC	PVBTMAC Monomeric Units	POEGMA Monomeric Units
PVBTMAC	40,000	-	100	-	-
POEGMA-*b*-PVBTMAC	4200	18,200	19	20	36

**Table 2:** DLS and ELS results for the POEGMA-*b*-PVBTMAC copolymer and POEGMA-*b*-PVBTMAC + 0.5 mg CoFe_2_O_4_ complexes in aqueous solutions.

Sample	*R_h_* (nm)	PDI	*ζ_pot_* (mV)
POEGMA-*b*-PVBTMAC_C = 5 × 10^−4^ g/ml	3/108	0.625	+20
POEGMA-*b*-PVBTMAC + 0.5 mg CoFe_2_O_4_	139	0.226	+14

**Table 3:** DLS results for the POEGMA-*b*-PVBTMAC copolymer, MNPs–hybrid complexes and magnetopolyplexes (*N*/*P* = 4) at 0.01 M NaCl.

Sample	*R_h_* (nm)	PDI	Int (kHz)
POEGMA-*b*-PVBTMAC_C = 5 × 10^−4^ g/ml	4/97/469	0.5	337
POEGMA-*b*-PVBTMAC + 0.5 mg CoFe_2_O_4_	300	0.340	714
POEGMA-*b*-PVBTMAC + 0.5 mg CoFe_2_O_4_ + DNA	283	0.295	2143

## Supplementary Materials

The following are available online at https://www.mdpi.com/2073-4360/12/6/1283/s1. Table S1: DLS at 90° and ELS characteristics of the CoFe_2_O_4_ nanoparticles; Table S2: Magnetic properties of the CoFe_2_O_4_ magnetic nanoparticles; Table S3: DLS characteristics of the POEGMA-*b*-PVBTMAC copolymer at C = 10^−3^ g/mL in aqueous solutions at 90° from cumulants; Figure S1: Size distribution from Contin for the hydrophilic CoFe_2_O_4_ nanoparticles at 90°; Figure S2: XRD pattern of CoFe_2_O_4_ nanoparticles; Figure S3: Hysteresis loops for CoFe_2_O_4_ magnetic nanoparticles; Figure S4: Size distribution from Contin for the POEGMA-*b*-PVBTMAC_C = 10^−3^ g/mL diblock at 90° in water; Figure S5: R_h_ and scattered intensity as a function of ionic strength ([NaCl]) for POEGMA-*b*-PVBTMAC at C = 10^−3^ g/mL; Figure S6: R_h_ and scattered intensity as a function of ([BSA]) for POEGMA-*b*-PVBTMAC + 0.5 mg CoFe_2_O_4_ at 90°; Figure S7: UV spectra of free DNA, POEGMA-*b*-PVBTMAC copolymer and POEGMA-*b*-PVBTMAC + MNPs + DNA hybrid polyplexes at *N/P* ratios in the 1–7 range; Figure S8: Cryo-TEM micrographs (a and b) of the POEGMA-*b*-PVBTMAC + 1 mg CoFe_2_O_4_ complexes.
