# Supplementary material for: Poly[oligo(ethylene glycol) methacrylate]-b-poly[(vinyl benzyl trimethylammonium chloride)] Based Multifunctional Hybrid Nanostructures Encapsulating Magnetic Nanoparticles and DNA
Source: Polymers (Basel). 2020 Jun 3;12(6):1283. doi: 10.3390/polym12061283 (PMC7362237; doi:10.3390/polym12061283)
Supplement: Supplementary file 1 [file polymers-12-01283-s001.pdf]

polymers MDPI

*Supplementary Material*

# Poly[oligo(ethylene glycol) methacrylate]-b-poly[(vinyl benzyl trimethylammonium chloride)] based multifunctional hybrid nanostructures encapsulating magnetic nanoparticles and DNA

**Angeliki Chroni [1], Aleksander Forys [2], Barbara Trzebicka [2], Adam Alemayehu [3], Václav Tyrpekl [3] and Stergios Pispas [1,*]**

[1] Theoretical and Physical Chemistry Institute, National Hellenic Research Foundation, 48 Vassileos Constantinou Avenue, 11635 Athens, Greece; angelikechrone@gmail.com
[2] Centre of Polymer and Carbon Materials, Polish Academy of Sciences, 34 ul. M. Curie-Skłodowskiej, 41-819 Zabrze, Poland; aforys@cmpw-pan.edu.pl; barbara.trzebicka@cmpw-pan.edu.pl
[3] Department of Inorganic Chemistry, Charles University in Prague, Faculty of Science, Albertov 6, 128 43 Praha 2; e-mail-Adam; vaclav.tyrpekl@natur.cuni.cz
* Correspondence: pispas@eie.gr; Tel.: +30-210-727-3824

## 3. Results and Discussion

### *3.2. Characterization of $CoFe_2O_4$ nanoparticles*

DLS, ELS and XRD measurements were performed to assess the size, the surface potential and the crystalline structure of the $CoFe_2O_4$ nanoparticles. The hydrodynamic radius ($R_h$), size polydispersity index (PDI) and zeta potential ($\zeta_{pot}$) values of the $CoFe_2O_4$ nanoparticles are gathered in Table S1. According to DLS measurements, the negatively charged $CoFe_2O_4$ nanoparticles (as seen from $\zeta_{pot}$ value in Table S1) form nanoparticles of 12 nm and aggregates of 70 nm in aqueous milieu. Particularly, the size distribution plot from Contin analysis for the $CoFe_2O_4$ nanoparticles in Figure S1 depicts two different intensity peaks with the first, at 12 nm, being probably attributed to the actual size of the nanoparticles and the second, at 70 nm, to the aggregates formed in aqueous solutions.

**Table S1.** DLS at 90° and ELS characteristics of the $CoFe_2O_4$ nanoparticles.

| Sample | $R_h$ (nm) | PDI | $\zeta_{pot}$ (mV) |
|---|---|---|---|
| $CoFe_2O_4$ NPs | 12 / 70 | 0.273 | -32 |

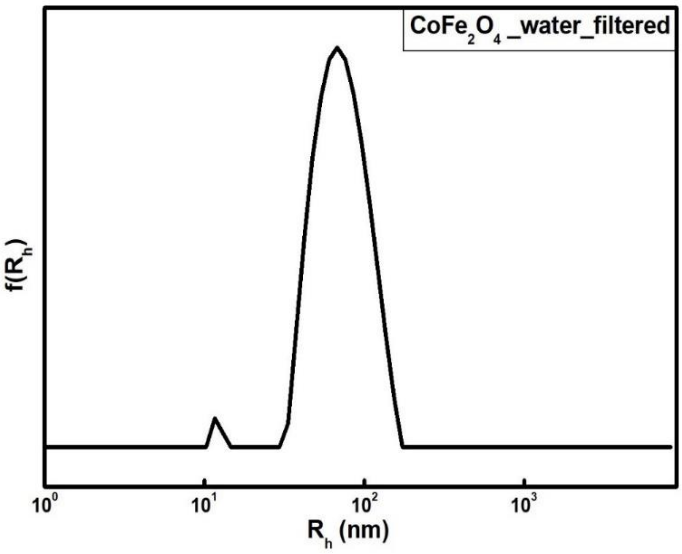


**Figure S1**. Size distribution from Contin for the hydrophilic $CoFe_2O_4$ nanoparticles at 90°.

The crystalline structure of $CoFe_2O_4$ was characterized by XRD. The XRD pattern in Figure S2 shows the strong reflections, namely (220), (311), (222), (400), (422), (511) and (440). The analysis of XRD pattern confirms the single-phase cubic spinel structure that corresponds to the f.c.c type and is consistent with the standard data of $CoFe_2O_4$ (JCPDS no. 79-1744) and literature. [1]

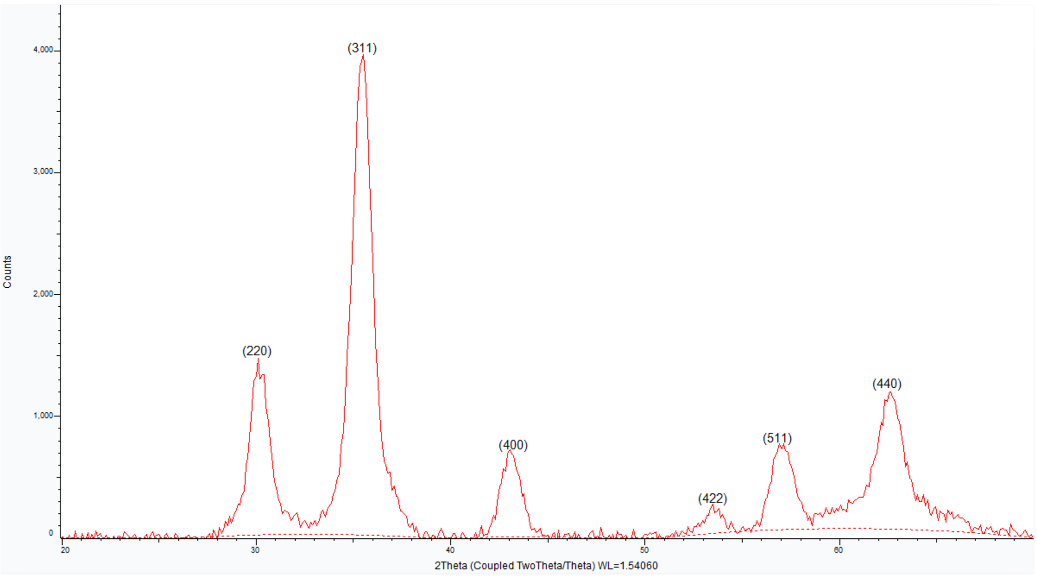


**Figure S2**. XRD pattern of $CoFe_2O_4$ nanoparticles.

*3.3. Physicochemical characterization of the POEGMA-b-PVBTMAC copolymer*

The $R_h$ and PDI values of the formed POEGMA-b-PVBTMAC nanostructures at a solution concentration of $10^{-3}$ g/ml in aqueous media are presented in Table S2, at pH=7 and 25°C. The size distribution from Contin analysis in Figure S3, appears symmetrical and almost monomodal revealing copolymer unimers of 4 nm and copolymer aggregates of 111 nm.

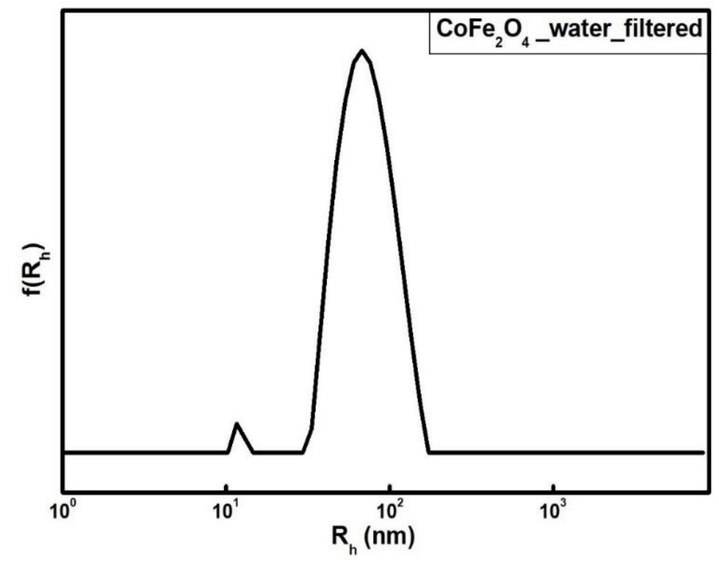


**Figure S1.** Size distribution from Contin for the POEGMA-b-PVBTMAC C=10-3 g/ml diblock at 90° in water.

**Table S1:** DLS characteristics of the POEGMA-b-PVBTMAC copolymer at $C=10^{-3}$ g/ml in aqueous solutions at 90° from cumulants.

| Sample | $R_h$ (nm) | PDI |
|---|---|---|
| POEGMA-b-PVBTMAC $C=10^{-3}$ g/ml | 111 | 0,392 |

*3.4. Effect of Solution Ionic Strength on the polyelectrolyte copolymer behavior*

The addition of NaCl in aqueous polyelectrolyte copolymer solutions often elicits significant changes in polymer solubility and size of the polymeric chains. The DLS measurements of the POEGMA-b-PVBTMAC copolymer were conducted at $10^{-3}$ g/ml. The dependence of $R_h$ and scattered intensity on the NaCl concentration is depicted in Figure S4 for the POEGMA-b-PVBTMAC copolymer. A significant growth in the diameters of POEGMA-b-PVBTMAC aggregates is observed, as $R_h$ increases with the gradual addition of salt, indicating the formation of aggregates of constant size above 0.1 M NaCl. A gradual increase in the mass of the aggregates is noticed as ionic strength increases from 0 to 0.03 M, followed by a secondary aggregation until scattered intensity reaches a plateu above 0.3 M NaCl.

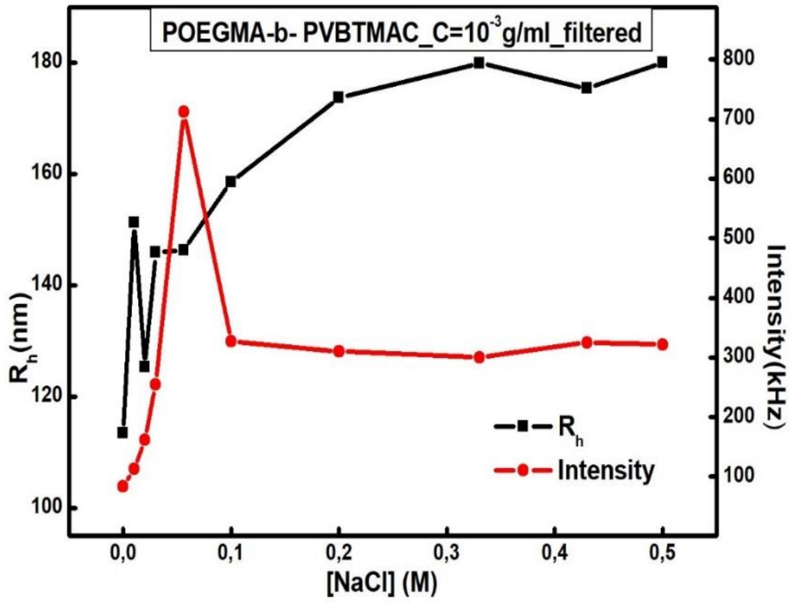


**Figure S4.** $R_h$ and scattered intensity as a function of ionic strength ([NaCl]) for POEGMA-b-PVBTMAC at $C=10^{-3}$ g/ml.

*3.6. Interaction of BSA with the diblock/MNPs hybrid complexes*

An examination of the interaction between MNPs-hybrid complexes with bovine serum albumin (BSA) provides a deeper understanding of their potential applications in biological environments such as blood serum. The BSA interaction with the hybrid nanostructures was investigated by titration with BSA ($1x10^{-3}$ g/ml stock solution concentration) in 0.01M NaCl solution and subsequently by DLS measurements. The stock solution of the POEGMA-b-PVBTMAC+0.5mg $CoFe_2O_4$ complexes was prepared at a concentration of $5x10^{-4}$ g/ml in 0.01M NaCl solution. In Figure S5, the dependence of $R_h$ and scattered intensity on BSA concentration is depicted for the MNPs-hybrid complexes. The significant decrease in scattered intensity, Figure S5, reveals a decrease in the mass of the hybrid complexes as the concentration of BSA rises from 0 to $8x10^{-4}$ g/ml accompanied by relatively stable $R_h$ values, indicating swelling of the hybrid complexes after the initial addition of BSA. Apparently, BSA interactions with the POEGMA-b-PVBTMAC+0.5mg $CoFe_2O_4$ complexes elicited partial disintegration of the MNPs-hybrid complexes, judging from the decrease in scattered intensity.

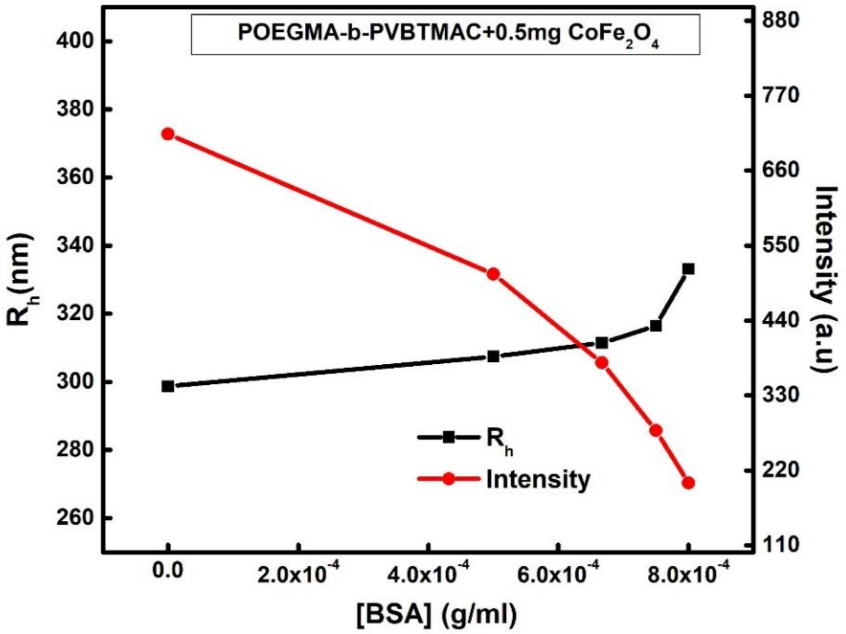


**Figure S5.** $R_h$ and scattered intensity as a function of ([BSA]) for POEGMA-b-PVBTMAC+0.5mg $CoFe_2O_4$ at 0.01 M NaCl.

### *3.7. Electrostatic complexation of POEGMA-b-PVBTMAC copolymer with Magnetic Nanoparticles and DNA*

Some extra evidence for the successful copolymer complexation firstly with MNPs and secondly with DNA is given by UV–Vis measurements. Only a few conclusions can be drawn regarding Figure S6, as the POEGMA-b-PVBTMAC copolymer exhibited multiple peaks in the UV spectra (222 nm, 264 nm and 318 nm) and the interpretation of the other UV peaks (presented in the case of magnetopolyplexes) become quite challenging. However, the increase in the absorbance at 450 nm is related to the presence of MNPs at all N/P ratios and not of the copolymers. Free DNA in Figure S6 exhibits a characteristic absorption peak at 260 nm which is fairly consistent with the literature.[2,3] As it can be seen from Figure S6, the intensity of the absorption peak at 260 nm decreases while magnetopolyplexes are formed with the positive charged polymer chains.

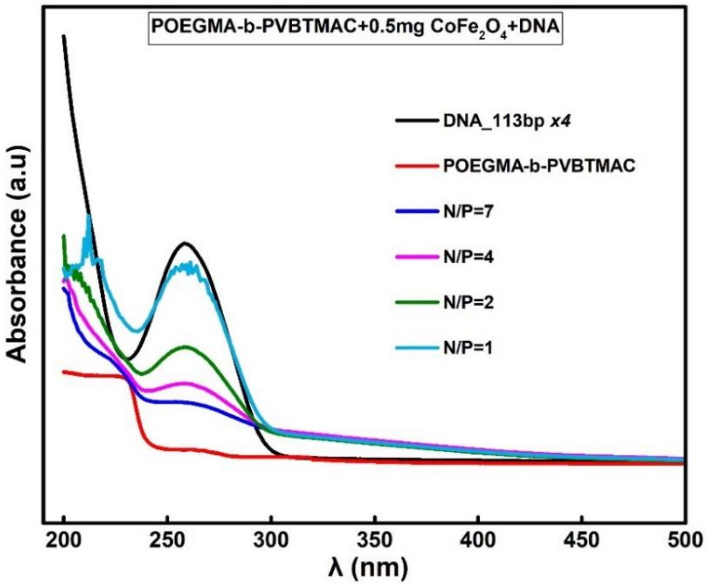


**Figure S6.** UV spectra of free DNA, POEGMA-b-PVBTMAC copolymer and POEGMA-b-PVBTMAC+MNPs+DNA hybrid polyplexes at N/P ratios in the 1-7 range.

*3.10. Cryogenic Transmission Electron Microscopy of the MNPs-hybrid complexes and DNA-hybrid polyplexes*

Cryo-TEM experiments were conducted for POEGMA-b-PVBTMAC+1mg $CoFe_2O_4$ complexes to have a more complete picture of the morphology of the iron oxide NPs. The measurements were conducted at $C_{polymer}$ = 5x10$^{-4}$ g/ml and pH 7. The micrographs in Figure S7, display one type of spherical, coagulated, nanoparticles (4-15 nm) while no substantial changes in their morphology is observed as the quantity of $CoFe_2O_4$ NPs increases.

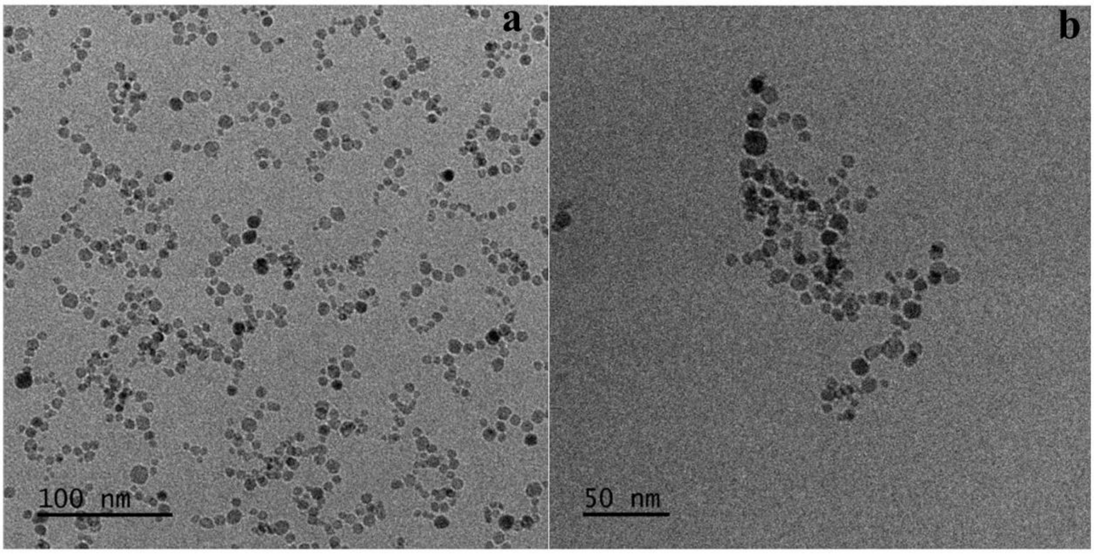


**Figure S7.** Cryo-TEM micrographs (a and b) of the POEGMA-b-PVBTMAC+1mg $CoFe_2O_4$ complexes.

## References

1. Zhao, S.; Ma, D. Preparation of CoFe. *J. Nanomater.* **2010**, *2010*.
2. Dey, D.; Kumar, S.; Banerjee, R.; Maiti, S.; Dhara, D. Polyplex formation between PEGylated linear cationic block copolymers and DNA: equilibrium and kinetic studies. *J. Phys. Chem. B* **2014**, *118*, 7012-7025.
3. Kwon, Y.-W.; Choi, D.H.; Jin, J.-I. Optical, electro-optic and optoelectronic properties of natural and chemically modified DNAs. *Polym. J.* **2012**, *44*, 1191-1208.

© 2020 by the authors. Submitted for possible open access publication under the terms and conditions of the Creative Commons Attribution (CC BY) license (http://creativecommons.org/licenses/by/4.0/).
